# Population variation in brain size of nine-spined sticklebacks (*Pungitius pungitius*) - local adaptation or environmentally induced variation?

**DOI:** 10.1186/1471-2148-11-75

**Published:** 2011-03-24

**Authors:** Abigél Gonda, Gábor Herczeg, Juha Merilä

**Affiliations:** 1 Ecological Genetics Research Unit, Department of Biosciences, University of Helsinki, PO Box 65, FI-00014, Helsinki, Finland

## Abstract

**Background:**

Most evolutionary studies on the size of brains and different parts of the brain have relied on interspecific comparisons, and have uncovered correlations between brain architecture and various ecological, behavioural and life-history traits. Yet, similar intraspecific studies are rare, despite the fact that they could better determine how selection and phenotypic plasticity influence brain architecture. We investigated the variation in brain size and structure in wild-caught nine-spined sticklebacks (*Pungitius pungitius*) from eight populations, representing marine, lake, and pond habitats, and compared them to data from a previous common garden study from a smaller number of populations.

**Results:**

Brain size scaled hypo-allometrically with body size, irrespective of population origin, with a common slope of 0.5. Both absolute and relative brain size, as well as relative telencephalon, optic tectum and cerebellum size, differed significantly among the populations. Further, absolute and relative brain sizes were larger in pond than in marine populations, while the telencephalon tended to be larger in marine than in pond populations. These findings are partly incongruent with previous common garden results. A direct comparison between wild and common garden fish from the same populations revealed a habitat-specific effect: pond fish had relatively smaller brains in a controlled environment than in the wild, while marine fish were similar. All brain parts were smaller in the laboratory than in the wild, irrespective of population origin.

**Conclusion:**

Our results indicate that variation among populations is large, both in terms of brain size and in the size of separate brain parts in wild nine-spined sticklebacks. However, the incongruence between the wild and common garden patterns suggests that much of the population variation found in the wild may be attributable to environmentally induced phenotypic plasticity. Given that the brain is among the most plastic organs in general, the results emphasize the view that common garden data are required to draw firm evolutionary conclusions from patterns of brain size variability in the wild.

## Background

During the past few decades, studies on diverse taxa have demonstrated that both absolute and relative brain size, as well as absolute and relative sizes of different brain parts, are highly variable and correlate with several environmental factors [mammals e.g. [1-3], birds e.g. [4,5] and fishes e.g. [6,7]]. Most of these studies, which form the basis of our current knowledge about brain size evolution, have used correlative approaches at the interspecific level. However, several recent studies have investigated differences in brain architecture among populations of the same species [8-17]. By using interpopulation comparisons, microevolutionary processes can be investigated explicitly because most populations are likely to be found in the environment that actually shaped their brains. This is an unlikely situation in the case of comparisons based on species, which might have gone through adaptive divergence after splitting from common ancestor. An additional benefit from interpopulation comparisons as compared to interspecific comparisons is that the former allow adopting approaches to separate the relative roles of genetic drift and natural selection on observed differentiation [e.g. [18,19]]. Further, even fewer studies have compared brains of individuals from different populations reared under standardized settings to exclude the possible effects of phenotypic plasticity [but see: [10,12]]. This is surprising considering the fact that phenotypic plasticity in overall brain size, in addition to the size of different brain regions, has often been demonstrated [e.g. seasonality: [20,21]; spatial learning: [22,23], environmental heterogeneity: [24,25]]. Moreover, how this plasticity might influence population and species comparisons in terms of neural architecture has yet to be explored. Therefore, direct comparisons of patterns based on data collected from wild populations with those based on data from standardized common garden settings are needed to establish if any evolutionary inferences can be made from wild collected data in such a highly plastic organ as the brain.

The nine-spined stickleback (*Pungitius pungitius*) is an excellent model species for intraspecific comparative studies and exploring adaptive divergence. It occupies markedly different habitats, ranging from marine environments through large lakes to isolated ponds wherein they are often the only fish species present [e.g. [26]]. Hence, large differences can be found both in biotic (e.g. diversity of prey, competitors and predators) and abiotic (e.g. habitat structure) habitat components. These differences are expected to impose different selection pressures on complex behaviours and memory, and thus, also on the neural architecture. This is especially true in light of the high energetic costs of developing and maintaining large brains [27]. Our recent studies, utilizing common garden reared nine-spined sticklebacks, have demonstrated genetically-based and habitat-related divergence in (i) size of different brain parts [12] and (ii) brain plasticity in response to the social environment [13]. However, patterns found in the wild have not been reported, and the fit between patterns of variation in common garden and wild collected data has never been tested.

Brain size scales allometrically with body size, both on ontogenetic and on evolutionary scales [e.g. [28-32]]. The slope of the allometric relationship between brain size to body size (both variables plotted on a logarithmic scale) is higher in prenatal than adult stages in mammals [28]. Furthermore, the slope of this relationship tends to be steeper at higher taxonomic ranks [ca. 0.75 across mammalian orders; e.g. [29,30]] compared to closely related species, or in intraspecific comparisons (ca. 0.2-0.5; [31,32]). Although some intraspecific studies in brain-body size allometry exist [e.g. [33]], only very few investigations have been conducted within a single vertebrate species, perhaps due to a lack of sufficient within-species size variation among adults. Since nine-spined sticklebacks living in ponds have repeatedly evolved into giants [34,35], the species (representing tenfold body weight differences between adult individuals) also provides an excellent model to study intraspecific brain-body size allometry.

**Figure 1:**
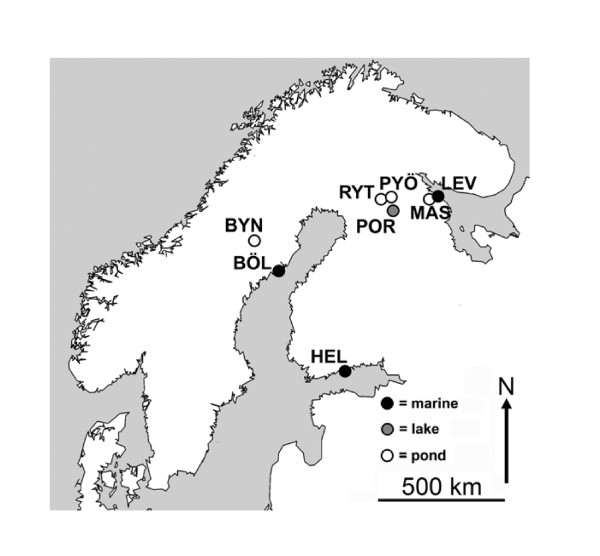
**Map of the sampling localities**. BÖL = Bölesviken, Baltic Sea, Sweden; HEL = Helsinki, Baltic Sea, Finland; LEV = Levin Navolok Bay, White Sea, Russia; POR = Porontima, Finland; BYN = Bynästjärnen, Sweden; PYÖ = Pyöreälampi, Finland; RYT = Rytilampi, Finland; MAS = Mashinnoje, Russia.

Our aim was to explore population divergence in brain size and in the size of different parts of the brain (*viz*. telencephalon, optic tectum, cerebellum, hypothalamus) in wild-caught nine-spined sticklebacks from different habitats, and to compare the observed patterns with previously reported common garden results [12]. We sampled fish from eight Fennoscandian populations (Figure 1) originating from three habitat types (*viz*. marine, lake and pond environments) to test (i) for differences in the size of the brain and different brain parts among wild populations, and (ii) whether observed differences were habitat specific. Furthermore, to establish whether data collected from the wild can be used for evolutionary inference, (iii) we tested whether data collected from the wild and common garden experiments for fish originating from the same populations are concurrent, and if not, (iv) whether observed differences are population- or habitat-specific. We expected that the higher biotic and abiotic variability of marine and lake environments as compared to pond environments have selected for relatively large brains. We also expected to find habitat-dependent differences in brain parts important in perception, learning and spatial memory, and that the stimulus-poor laboratory environment would reduce the brains of common garden fish compared to their wild conspecifics. We also explored the brain-body size allometric relationship in fish from different populations, expecting hypoallometry with a relatively shallow slope (<0.5).

## Results

### Variation in absolute brain size

**Figure 2:**
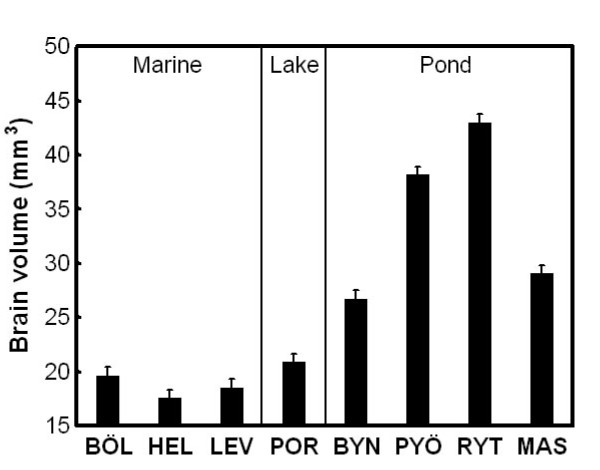
**Population variation in absolute brain size in nine-spined sticklebacks**. Means ± SE are shown. For population abbreviations, see Fig. 1.

Dissected brains were fixed and photographed under standardized conditions. Absolute brain size was estimated from measurements taken from digital photographs (dorsal, lateral and ventral views) by using the ellipsoid model [12,36]. General Linear Model (GLM) results revealed a significant population effect in absolute brain size (*F*_7, 112 _= 153.68, *P *< 0.001). Average brain sizes of marine and lake fish were similar, but smaller than those of pond fish, the latter also being highly variable (Figure 2). General Linear Mixed Model (GLMM) analyses revealed a significant habitat (*F*_1, 5 _= 11.84, *P *= 0.018) and non-significant population within habitat effect (*Z *= 1.55, *P *= 0.12). Pond fish had brains almost twice as large as marine fish (Least Squares [LS] mean ± Standard Error [SE]: marine = 18.59 ± 3.43 mm^3^; pond = 34.22 ± 2.97 mm^3^).

**Figure 3:**
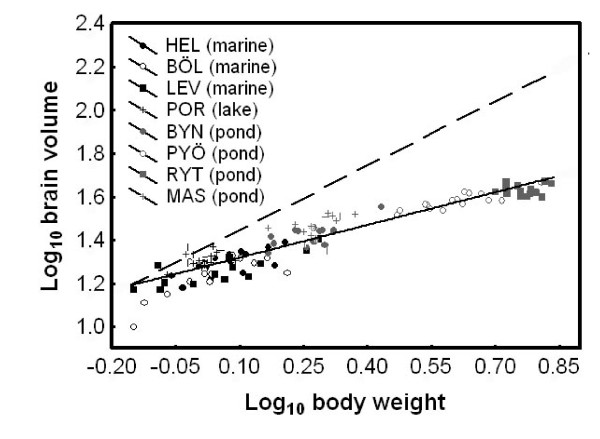
**Allometric relationship between brain size and body size in nine-spined sticklebacks**. Since we detected no significant population-specific relationship, only the common slope (solid line; *β *= 0.50) is shown. The dashed line denotes isometry. For population abbreviations, see Fig. 1.

Brain size co-varied with body weight, but independently of population origin and standard length (GLM; population: *F*_7, 96 _= 1.50, *P *= 0.18; log body weight: *F*_1, 96 _= 7.63, *P *= 0.007; log standard length: *F*_7, 96 _= 0.52, *P *= 0.47; population × log body weight: *F*_7, 96 _= 0.79, *P *= 0.60; population × log standard length: *F*_7, 96 _= 1.48, *P *= 0.18). The log brain size - log body weight regression revealed hypoallometry (i.e. both *β *= 0 and *β *= 1 were rejected: *R*^2 ^= 0.88, *β *= 0.50, SE[*β*] = 0.02, *P *< 0.001; Figure 3), indicating that brain size increased at half the rate of body size.

### Variation in relative brain size and brain part size

**Figure 4:**
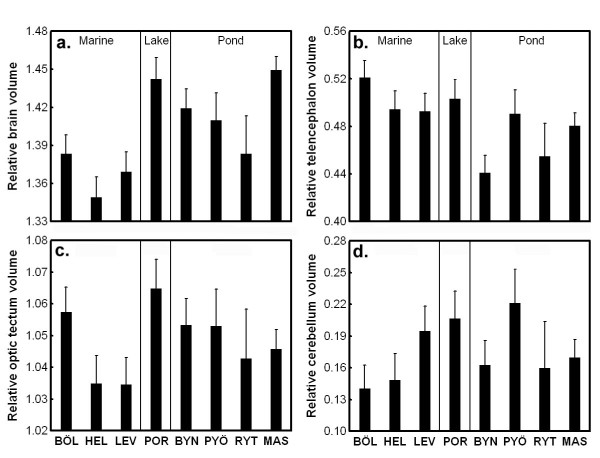
**Population variation in relative brain size and relative size of different brain parts**. Least Squares means ± SE are shown. Relative brain size is corrected for standard length and body weight while relative size of different brain parts are corrected for standard length, body weight and brain volume. For population abbreviations, see Fig.1.

To study variation in relative brain size, we corrected the absolute brain size estimates to body length and body weight. We detected significant differences in relative brain size both at the habitat (GLMM; habitat: *F*_1, 6.89 _= 10.38, *P *= 0.015; log standard length: *F*_1, 57.02 _= 0.27, *P *= 0.54; log body weight: *F*_1, 93.48 _= 28.82, *P *< 0.001; population [habitat]: *Z *= 0.95, *P *= 0.34), and population level (GLM; population: *F*_7, 110 _= 10.79, *P *< 0.001; log standard length: *F*_1, 110 _= 0.02, *P *= 0.89; log body weight: *F*_1, 110 _= 30.14, *P *< 0.001). Pond (and the single lake) populations had larger relative brain sizes than marine populations (Figure 4a).

The sizes of different brain parts (telencephalon, optic tectum, cerebellum, hypothalamus) were also estimated from the digital photographs using the ellipsoid model [12,36]. We did not consider absolute size, but corrected our estimates with body length, body weight and absolute brain size. The multivariate GLM revealed a significant population effect on the relative sizes of different brain parts (population: Wilks' λ_28, 380 _= 0.53, *P *< 0.001; log body weight: Wilks' λ_4, 105 _= 0.91, *P *= 0.043; log standard length: Wilks' λ_4, 105 _= 0.95, *P *= 0.024; log brain volume: Wilks' λ_4, 105 _= 0.15, *P *< 0.001). Subsequent univariate tests indicated significant population differences in relative telencephalon (*F*_7, 108 _= 3.53, *P *= 0.002), optic tectum (*F*_7, 108 _= 2.81, *P *= 0.010), and cerebellum (*F*_7, 108 _= 2.59, *P *= 0.016) sizes, but not in hypothalamus (*F*_7, 108 _= 1.47, *P *= 0.18) size (Figure 4b-d). Our GLMMs revealed that neither optic tectum (habitat: *F*_1, 10.17 _= 0.44, *P *= 0.52; log standard length: *F*_1, 59.62 _= 0.26, *P *= 0.61; log body weight: *F*_1, 88.93 _= 0.50, *P *= 0.48; log brain volume: *F*_1, 99.99 _= 262.72, *P *< 0.001; population[habitat]: *Z *= 1.01, *P *= 0.31) nor cerebellum (habitat: *F*_1, 10.02 _= 0.03, *P *= 0.87; log standard length: *F*_1, 64.12 _= 0.35, *P *= 0.056; log body weight: *F*_1, 93.92 _= 7.16, *P *= 0.009; log brain volume: *F*_1, 99.91 _= 32.39, *P *< 0.001; population[habitat]: *Z *= 1.12, *P *= 0.26) showed significant habitat-dependency in their relative size. However, in the case of telencephalon, the habitat effect approached significance (habitat: *F*_1, 9.08 _= 3.95, *P *= 0.078; log standard length: *F*_1, 61.39 _= 5.11, *P *= 0.027; log body weight: *F*_1, 93.10 _= 1.37, *P *= 0.24; log brain volume: *F*_1, 99.91 _= 122.53, *P *< 0.001; population [habitat]: *Z *= 1.05, *P *= 0.29). Marine fish tended to have larger telencephala than pond fish, while no systematic trend could be observed in the other brain parts (Figure 4b-d).

### Comparison of wild and common garden brains

**Figure 5:**
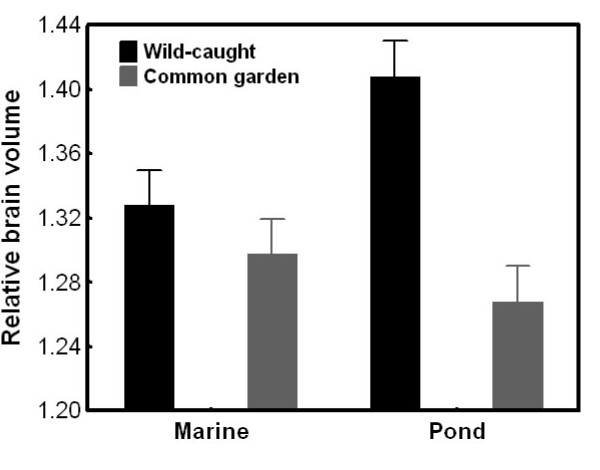
**Habitat specific differences in relative brain size between wild-caught and common garden nine-spined sticklebacks**. Least Squares means ± SE are shown. Relative brain size is corrected for standard length and body weight.

We compared relative brain size and relative brain part size (see above) of fish from two marine (Helsinki, Baltic Sea and Levin Navolok Bay, White Sea; Figure 1) and two pond (Bynästjärnen, Sweden and Pyöreälampi, Finland; Figure 1) populations to data from the same populations reared in a common garden experiment [12]. Fish origin (i.e. wild-caught *vs*. common garden) had a habitat specific effect on relative brain size (GLMM; origin: *F*_1, 113.11 _= 70.99, *P *< 0.001; habitat: *F*_1, 2.49 _= 0.65, *P *= 0.49; origin × habitat: *F*_1, 113.83 _= 38.36, *P *< 0.001; log standard length: *F*_1, 105.99 _= 5.75, *P *= 0.018; log body weight: *F*_1, 113.95 _= 42.12, *P *< 0.001; population[habitat]: *Z *= 0.84, *P *= 0.40). The population-level GLM supported this result. It revealed a population-specific origin effect (origin: *F*_1, 110 _= 70.41, *P *< 0.001; population: *F*_3, 110 _= 5.55, *P *= 0.001; origin × population: *F*_3, 110 _= 14.51, *P *< 0.001; log standard length: *F*_1, 110 _= 9.56, *P *= 0.003; log body weight: *F*_1, 110 _= 46.65, *P *< 0.001). Relative brain size was similar for wild-caught and common garden marine fish, whereas pond fish had relatively larger brains in the wild than in the laboratory (Figure 5).

**Figure 6:**
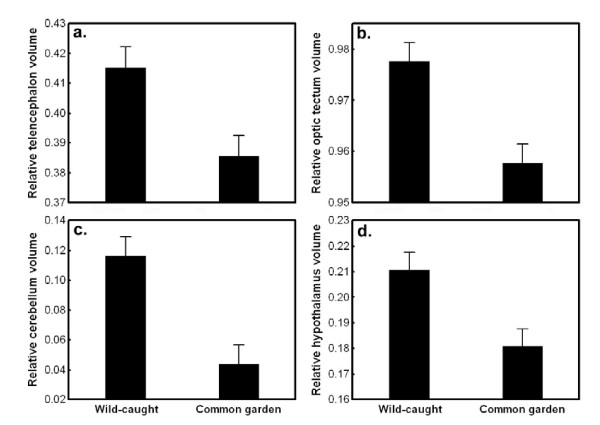
**The relative size of different brain parts in wild-caught and common garden nine-spined sticklebacks**. Least Squares means ± SE are shown. Relative size of different brain parts are corrected for standard length, body weight and brain volume.

A multivariate GLM revealed significant, simple effects of population and origin on the relative size of brain parts, but no interaction between variables (origin: Wilks' λ_4, 106 _= 0.74, *P *< 0.001; population: Wilks' λ_12, 280.7 _= 0.68, *P *< 0.001; origin × population: Wilks' λ_12, 280.7 _= 0.91, *P *= 0.57; log standard length: Wilks' λ_4, 106 _= 0.93, *P *= 0.12; log body weight: Wilks' λ_4, 106 _= 0.89, *P *= 0.012; log brain volume: Wilks' λ_4, 106 _= 0.107, *P *< 0.001). All brain parts were affected by origin, as revealed by the subsequent univariate tests (5.91 <*F*_1, 109 _< 10.27, 0.002 <*P *< 0.017). Wild-caught fish had relatively larger telencephalon, optic tectum, cerebellum and hypothalamus volumes than their common garden reared conspecifics (Figure 6a-d).

## Discussion

We showed that there is large variation in absolute brain volume, relative brain volume and relative volume of the telencephalon, optic tectum and cerebellum across wild nine-spined stickleback populations. Brain size patterns in the wild show habitat specificity both in absolute and relative scales: pond fish have larger brains than marine fish. Further, we found a marginally significant trend in the relative telencephalon size: marine fish tend to have larger telencephala than pond fish. The hypoallometric relationship between brain size and body size (slope = 0.5) is in accordance with a previous study on tropical fish [37]. We also found that wild-caught pond fish have larger brains than laboratory-reared pond fish, whereas no differences were observed between wild-caught and laboratory-reared marine conspecifics. The relative sizes of all brain parts were smaller in common garden than in the wild in all populations. These findings indicate that even though large brain size and brain part size variation exist in the wild, both in absolute and relative terms, patterns in nature may differ from those gathered in a standardized common garden and in some cases even in a habitat-dependent way. This strongly suggests that environmental effects on brain development can obscure and confound evolutionary inference based on purely phenotypic data collected from the wild. Hence, our results under-line the importance of not basing evolutionary inference on phenotypic patterns of brain size variation unless the environmental sources of variation have been controlled for - a point reinforced by other studies focussing on differentiation in morphological and life history traits [38-40].

We found large habitat-specific population variation in absolute brain size: all marine populations (and a single lake population) had similarly sized brains that were nearly twofold smaller than those of pond fish. Within the pond habitat, there was large variation in average brain size. Although most studies have investigated only relative brain size variation (correcting brain size for variation in body size), absolute brain size can also account for differences in behaviour and/or habitat use. This is evident in comparisons of closely related species [see e.g. [32]], as the brains of these species tend to be more similar than those of distant taxa. Indeed, absolute brain size variation is routinely utilized in studies of primate and human evolution [e.g. [41]]. In general, increased brain size is attributable to an increase in neuron number, and not in neuron size [32]. Further, increases in absolute brain size result in decreased proportional connectivity [32]. Obviously, larger bodies need larger brains to be controlled at a similar level [32]. Hence, it is not surprising that pond populations that have evolved to giants [34,35] have also much larger brains than smaller sized marine or lake populations.

Previous findings have demonstrated a shallower hypoallometric slope at the intraspecific level and among closely related species than across broader taxonomic groups (mammals, intraspecific: 0.2-0.4, broad interspecific: 0.66, [28]; fishes, intraspecific: 0.44, intrafamiliar: 0.5, broad interspecific: 0.66, [37]). In accordance with these results we found a hypoallometric relationship between brain and body size with a slope = 0.5. In mammals, it has been demonstrated that on a broad evolutionary time scale, there has been greater net directional selection on brain size than on body size, while the short-term differentiation in brain *vs*. body size in closely related mammalian taxa has resulted from directional selection acting mostly on body size with changes in brain sizes being largely correlated responses [32]. Further, Gonzalez-Voyer et al. [42] demonstrated in Tanganyikan cichlids that even strongly correlated traits, such as brain and body size, can evolve independently from each other, and that body size may be under stronger selection than brain size during adaptive radiation. In the case of the nine-spined stickleback system, habitat-dependent body size diversification has been demonstrated [34,35], and body weight differences among these recently differentiated populations can be tenfold. Hence, it seems feasible to suggest that the observed brain size divergence might have been a correlated response to body-size divergence.

To assess body-size-independent brain size trends, we also investigated brain size differences relative to body size. Similarly to results for absolute brain size, and in contrast to our expectations, pond sticklebacks had relatively larger brains than marine sticklebacks. Intraspecific variation in relative brain size and brain architecture also appears to be strongly correlated with different ecological factors and/or life history traits. For example, environmental harshness has been shown to correlate positively with the size and neuron number of the brain region linked with memory storage (hippocampus) in the black-capped chickadee, *Poecile atricapillus*, a food caching species for which good memory can be essential for survival [14]. Garamszegi & Eens [8] found a positive correlation between song length and repertoire size and both relative and absolute volumes of different song nuclei. By comparing two subspecies of the white-crowned sparrow, the migratory *Zonotrichia leucophrys gambelii *and non-migratory *Z. l. nuttalli*, Pravosudov et al. [43] found that migratory subspecies had larger hippocampus and more hippocampal neurons. Habitat-independent, genetically based intraspecific variation in brain architecture has also been found both in wild guppy (*Poecilia reticulata*) populations reared in common environments [11], and in laboratory lines of the medaka (*Oryzias latipes; *[10]).

Marine nine-spined sticklebacks are members of a diverse fish fauna, and as such, are faced with numerous predators and interspecific competitors. In contrast, pond fish communities are much simpler. Structural heterogeneity in the pond environment is also much lower than that found in marine environments. These environmental factors are all known to be important in shaping brain evolution. For instance, predation pressure has been shown to affect brain size evolution in Mallorcan bovids [44], diet affected brain size evolution of bats [45], both environmental complexity and social features sculpt the brain architecture of cichlid fish [36], while living in large and socially complex groups is the most accepted hypothesis for the evolution of the extremely large brain of humans [46]. Hence, we expected selective pressures stemming from predation, interspecific competition, and habitat complexity to result in relatively larger brains in marine populations. Moreover, assuming that body size divergence (pond fish > marine/lake fish [34,35]) preceded correlated brain size divergence, we also expected pond fish to have similar or smaller brains, in relative terms, than marine or lake fish. Our previous common garden experiment based on a subset of the populations used here revealed no habitat-dependence in relative brain size [12]. Therefore, the pattern found in the current study (pond fish > marine fish) is highly unlikely to be a result of selection on brain size itself. Further, while we found no habitat-dependence in the common garden setting, strong population differentiation in relative brain size in a habitat-independent way was detected (selective force unknown; [12]). Therefore, the plasticity resulting in the habitat-dependent wild *vs*. common garden difference cannot be habitat-specific itself. In a controlled laboratory experiment we found that group rearing had a negative effect on brain development in pond but not in marine fish [13]. Hence, the hypothesis that the aggressive, bold and antisocial pond fish [47,48] have larger relative brain sizes due to ontogenetic phenotypic plasticity as a response to fierce intraspecific competition must be rejected. Another possible explanation for larger relative brains in pond than in marine populations can be found from differences in ontogenetic allometry: pond fish living under negligible predation can become twice as old as marine fish [34], and an ontogenetic change in body *vs*. brain growth might explain this pattern. However, this issue requires further investigation.

Not only absolute and relative brain size, but also the relative size of different brain parts of nine-spined sticklebacks varied in the wild. Significant population differences were found in the relative sizes of the telencephalon, optic tectum and cerebellum. Further, we found marginally significant (*P *< 0.08) habitat-specificity in the relative size of the telencephalon, with marine fish tending to develop larger telencephala than pond fish. This is in accordance with results from our previous common garden study [12]. The telencephalon is larger in monogamous species, and shows a trend towards a positive correlation with rock size in the habitats in Tanganyikan lake cichlids [36], suggesting that both social and environmental heterogeneity may select for larger telencephalon. However, quite surprisingly, generalist limnetic populations of three-spined sticklebacks (*Gasterosteus aculeatus*) that use plankton as a main food source have larger telencephala than benthic-foraging populations of the same species as measured in samples from the wild [16]. The optic tectum is relatively larger in fish that prey on fish or other fast-moving prey, and clear water fishes develop larger optic tecta than species inhabiting turbid waters [6]. Cerebellum size correlates positively with the number of sympatric species in a fish community, and hypothalamus size is larger in monogamous than polygamous cichlids [36]. However, we did not find habitat specificity in the relative size of the optic tectum or the cerebellum, neither in the present, nor in the previous common garden study [12].

There are some incongruence between the present study and our previous work [12]. Here we found habitat-specific brain size divergence and population divergence in relative optic tectum size that was not seen in the common garden study. Only the habitat-dependence of relative telencephalon size found in the common garden study could be detected in the data from wild fish. A direct comparison between common garden and wild brains from the same populations revealed a habitat-dependent effect: pond (but not marine) fish had relatively larger brains in the wild than in the common garden. Further, the relative size of all brain parts was smaller in the laboratory than in the wild, perhaps due to a stimulus-poor environment during brain development. The most plausible explanation for the differences among common garden and wild data resides on phenotypic/ontogenetic plasticity in brain architecture. The potential for plastic responses to environmental heterogeneity is very high in fish [49-51]. Neurogenesis persists long into adulthood in fish [51-53] and contributes to lifelong growth of the brain. Hence, the fact that pond fish can live nearly twice as long as marine or lake fish may result in bias originating from plain ontogenetic plasticity or allometry. Furthermore, local random environmental variation may induce plasticity that could conceal genetic trends. Therefore, common garden studies seem to be of particular importance in studies of brain evolution. For instance, in this study system erroneous evolutionary conclusions could be drawn from the habitat-specificity (implying local adaptation) of relative brain size in the data from the wild given that observed differences cannot be reproduced under common garden conditions (showing that the differences are environmentally induced).

Finally, we showed that relative brain size and brain architecture are different between wild-caught and common garden sticklebacks from the same populations. The negative effect of domestication on brain size is well known both as a result of genetic adaptation and phenotypic plasticity [54,55]. In a recent paper, Burns et al. [56] demonstrated that laboratory rearing caused a significant decrease in the relative brain size of guppies (*P. reticulata*). Interestingly, we found that laboratory rearing had a negative effect on brain size in pond but not in marine nine-spined sticklebacks. The reason for this difference is unknown and warrants further investigations. We also found that all brain parts (corrected for both body and brain size) were smaller in common garden than in nature, a pattern congruent with general expectations. The reason for this can be a phenotypically plastic response to the comparatively stimulus poor laboratory environment.

## Conclusion

In summary, we found large variation both in absolute and relative brain size, and brain architecture, among nine-spined sticklebacks in the wild. However, the patterns differed markedly from those found previously under standardized common garden settings, being most probably a result of environmental or age effects prevailing in the wild. Further, we found that the difference between wild or common garden samples can be habitat specific. Considering the extreme plasticity of the fish brain, drawing evolutionary inference from wild-collected material alone can be challenging, and easily misleading. To understand brain size/structure variation in the wild, more intraspecific, common garden based studies, especially those that attempt to separate genetic and environmental contributions to brain development are needed.

## Methods

### Sampling and husbandry

Adult fish were collected from eight populations during May and June of 2007. Three habitat types were covered: marine samples came from Helsinki (Baltic Sea, Finland), Bölesviken (Baltic Sea, Sweden) and Levin Navolok (White Sea, Russia); Rytilampi (Finland), Pyöreälampi (Finland) and Bynästjärnen (Sweden) are isolated ponds, and Iso-Porontima (Finland) is a lake (Figure 1). This region started to deglaciate around 8000 years ago [e.g. [57]], thus, the populations are younger than this. Fish were collected with seine nets and minnow traps. After collection, they were moved to the aquaculture facilities of the University of Helsinki. Prior to taking brain measurements, fish were kept in standardized environment for approximately three months: temperature (14°C) and photoperiod (12 h light, 12 h dark) were held constant, and feeding (*ad libitum*) with bloodworms (Chironomidae sp.) was similar for all population groups. We note that the ca. three months common garden keeping (to standardize body condition, which is highly variable during spring in nature) might have caused some plastic responses induced by the artificial environment. However, this effect is highly unlikely to be profound. The experiment was conducted under license of the Animal Experiment Board in Finland, reference number: STH379A.

Sampled habitat types differed markedly, both in terms of biotic and abiotic aspects. In marine and lake habitats, nine-spined sticklebacks belong to a diverse fish community consisting of a large number of potential fish predators and interspecific competitors. Conversely, ponds lack predatory fish, and interspecific competition is absent (Rytilampi and Bynästjärnen), or negligible (Pyöreälampi; where a few, small-sized whitefish [*Coregonus lavaretus*] were recently introduced). While predation by aquatic insects and cannibalism at very early stages might occur in both habitats, there are two facts indicating large differences in predation caused mortality at later-than-fry stages: pond fish (i) show marked reduction in their defensive body armour (pelvic apparatus; [58]) and (ii) have a much longer lifespan than marine fish (6-7 years *vs*. 3-4 years; [34]). The structural complexity of the marine and large lake environment exceeded that of the study ponds which exhibit very simple structure (*viz*. negligible vegetation, and only a few rocks or fallen logs at the bottom of the pond). Although we did not quantify the abundance of nine-spined sticklebacks, it was evident from catch numbers and relative effort that population densities in ponds exceed those in the marine environment.

### Brain measurements

The entire procedure, from dissection through photography and measurement of whole brains and brain regions, followed exactly those outlined in Gonda et al. [12,13]. Fish (N = 15 per population) were euthanized with an overdose of MS 222 (tricaine methanesulfonate). Body weights were measured to the nearest 0.01 g with a digital balance and standard length (from the tip of the mouth to the end of the caudal peduncle) to the nearest 0.01 mm with digital callipers (for population variation in body weight and standard length see Additional file 1). Freshly dissected brains were fixed in 4% buffered formalin (0.1 M phosphate buffered saline) solution for 48 h. After fixation, digital photos were taken.

Width, height and length of the brain and four different parts of the brain - telencephalon, optic tectum, cerebellum and hypothalamus - were measured from the digital photographs using tpsDig 1.37 [59] software. They were defined as the greatest distance enclosed by the given structure. As the brains could not be cut off from the spinal cord at comparable positions in every individual, the end of the brain was defined as the perpendicular projection of the cerebellum on the medulla. We calculated the volume of the different brain parts according to the ellipsoid model [e.g. [60,36]]. Total brain volume was estimated with two different methods: first, with the equation of the ellipsoid model suggested by Pollen et al. [36]; second, we calculated brain volume by summing the volumes of the different parts. Both methods gave qualitatively similar results, thus, only the results from the ellipsoid model are reported. Repeatability (R) of the volume estimates was calculated from three repeated independent measurements of three independent photographs of a subsample of brains (N = 20). All volume variables were highly repeatable (R > 0.86, P < 0.001).

### Analyses

Absolute brain size was compared among populations using a General Linear Model (GLM) with brain volume as dependent variable and population as fixed factor. To test the habitat effect directly, we also ran a General Linear Mixed Model (GLMM) with brain volume as a dependent variable, habitat type (marine vs. pond) as a fixed effect, and population, nested within habitat type, as a random factor. Note that in this and the subsequent (see below) tests of habitat effects we excluded the single lake population and only compared three marine with four pond populations. To account for an allometric brain size - body size relationship, all metric variables were log_10 _(hereafter log) transformed. Because a GLM with log body weight as a dependent variable revealed population dependent patterns in the log standard length - log body weight relationship (population: *F*_7, 104 _= 13.02, *P *< 0.001; standard length: *F*_1, 104 _= 152.73, *P *< 0.001; population × standard length: *F*_7, 104 _= 14.56, *P *< 0.001), subsequent analyses of total brain size or brain part size included both log standard length and log body weight for size correction.

To study body - brain size allometry, we performed a GLM with log brain volume as the dependent variable, population as a fixed factor, and log standard length and log body weight as covariates, including factor × covariate interactions. We also ran a simplified GLM with only log body weight as a covariate, but the results remained qualitatively the same (data not shown). As only log body weight was significant in the model (see Results) the slope of the log brain size - log body weight correlation was determined by linear regression.

To assess relative brain size trends, we applied two approaches. First we ran a GLMM using log brain volume as the dependent variable, with habitat fixed and population nested within the habitat (random) factor, and log body weight and log standard length as covariates. Second, to compare populations directly, we ran a GLM with log brain volume as a dependent variable, population as a fixed factor and log body weight and log standard length as covariates. To compare the relative size of different brain parts, we ran a multivariate GLM with the brain parts as dependent variables, population as a fixed factor, and log body weight, log standard length and log brain volume as covariates. We note that random factors could not be properly computed in the multivariate context, and therefore, habitat effects could not be tested directly. In cases of a significant multivariate effect, related univariate tests were also performed. Upon significant univariate effect, we ran GLMMs testing for habitat effects with the given brain part as dependent variables, habitat as a fixed effect and population, nested within habitat, as a random factor, with log body weight, log standard length and log brain volume as covariates.

Finally, using data (N = 15 per population) from our previous common garden experiment [12] we compared a restricted set of populations for which we had data from both the wild and common garden. These populations were Helsinki (Baltic Sea, Finland), Levin Navolok Bay (White Sea, Russia), Bynästjärnen (pond, Sweden) and Pyöreälampi (pond, Finland). For these data we did not address absolute size. We compared relative brain volume by first running a GLMM with log brain volume as the dependent variable, habitat (marine *vs*. pond), origin (wild *vs*. common garden) and their interaction as fixed factors; population nested within habitat as a random factor, and log standard length and log body weight as covariates. Second, to compare populations, we ran a GLM with log brain volume as the dependent variable, population, origin and their interaction as fixed factors, and log standard length and log body weight as covariates. To compare the relative size of different brain parts, we ran a multivariate GLM with the log brain parts as dependent variables, population, origin and their interaction as fixed factors, and log standard length, log body weight and log brain volume as covariates. Upon significant multivariate effects, the related univariate tests were considered. Because we did not find significant population × origin interaction in the multivariate GLM (see Results), we did not address habitat × origin effects with GLMMs.

As in all GLMs and GLMMs testing for population or habitat divergence in relative brain volume or relative brain part volume the covariates were only used for correction, we did not include factor-covariate interactions. All statistical analyses were performed with the SPSS 18.0 for Windows package (SPSS Inc., Chicago, Illinois, USA).

## Authors' contributions

AG collected data for the study, dissected the brains, took photos of them, measured the sizes of the brains and the sizes of the different brain parts and participated in the design of the study and writing of the manuscript. GH performed the statistical analyses, participated in the design of the study and helped in the writing of the manuscript. JM participated in the design and coordination of the study and helped in the writing of the manuscript. All authors read and approved the final manuscript.

## Supplementary Material

Additional file 1 **Body size of the nine-spined sticklebacks (*Pungitius pungitius*) used in this study**. Standard length and body weight of the nine-spined stickleback (*Pungitius pungitius*) individuals used in the present study in the different populations. Mean ± SD and the minimum - maximum range are presented. Click here for file

## References

[B1] Harvey PH Clutton-Brock TH Mace GM Brain size and ecology in small mammals and primates Proc Natl Acad Sci USA 1980 77 4387 4389 10.1073/pnas.77.7.4387 PMC349840 6933492

[B2] Clutton-Brock TH Harvey PH Primates, brains and ecology J Zool 1980 190 309 323

[B3] Marino L A comparison of encephalization between Odontocete Cetaceans and anthropoid primates Brain Behav Evol 1998 51 230 238 10.1159/000006540 9553695

[B4] Day LB Westcott DA Olster DH Evolution of bower complexity and cerebellum size in bowerbirds Brain Behav Evol 2005 66 62 72 10.1159/000085048 15855743

[B5] Aviles JM Garamszegi LZ Egg rejection and brain size among potential hosts of the common cuckoo Ethology 2007 113 562 572

[B6] Kotrschal K Van Staaden MJ Huber R Fish brains: evolution and environmental relationships Rev Fish Biol Fisheries 1998 8 373 408

[B7] Gonzalez-Voyer A Winberg S Kolm N Social fishes and single mothers: brain evolution in African cichlids Proc R Soc B 2009 276 161 167 10.1098/rspb.2008.0979 PMC2614256 18796397

[B8] Garamszegi LZ Eens M Brain space for a learned task: strong intraspecific evidence for neural correlates of singing behavior in songbirds Brain Res Revs 2004 44 187 193 10.1016/j.brainresrev.2003.12.001 15003393

[B9] Karlen SJ Krubitzer L Phenotypic diversity is the cornerstone of evolution: Variation in cortical field size within Short Tailed Opossums J Comp Neur 2006 499 990 999 10.1002/cne.21156 17072834

[B10] Ishikawa Y Yoshimoto M Yamamoto N Ito H Different brain morphologies from different genotypes in a single teleost species, the medaka (*Oryzias latipes*) Brain Behav Evol 1999 53 2 9 10.1159/000006577 9858800

[B11] Burns JG Rodd H Hastiness, brain size and predation regime affect the performance of wild guppies in a spatial memory task Anim Behav 2008 76 911 922

[B12] Gonda A Herczeg G Merilä J Adaptive brain size divergence in nine-spined sticklebacks (*Pungitius pungitius*)? J Evol Biol 2009 22 1721 1726 10.1111/j.1420-9101.2009.01782.x 19549140

[B13] Gonda A Herczeg G Merilä J Habitat-dependent and -independent plastic responses to social environment in the nine-spined stickleback (*Pungitius pungitius*) brain Proc R Soc B 2009 276 2085 2092 10.1098/rspb.2009.0026 PMC2677246 19324759

[B14] Roth TC Pravosudov VV Hippocampal volumes and neuron numbers increase along a gradient of environmental harshness: a large-scale comparison Proc Roy Soc B 2009 276 401 405 10.1098/rspb.2008.1184 PMC2664346 18945667

[B15] Kolm N Gonzalez-Voyer A Brelin D Winberg S Evidence for small scale variation in the vertebrate brain: mating strategy and sex affect brain size and structure in wild brown trout (*Salmo trutta*) J Evol Biol 2009 22 2524 2531 10.1111/j.1420-9101.2009.01875.x 19878498

[B16] Park PJ Bell MA Variation of telencephalon morphology of the threespine stickleback (*Gasterosteus aculeatus*) in relation to inferred ecology J Evol Biol 2010 23 1261 1277 10.1111/j.1420-9101.2010.01987.x 20406344

[B17] Chrispo E Chapman LJ Geographic variation in phenotypic plasticity in response to dissolved oxygen in an African cichlid fish J Evol Biol 2010 23 2091 2103 10.1111/j.1420-9101.2010.02069.x 20722894

[B18] Merilä J Crnokrak P Comparison of genetic differentiation at marker loci and quantitative traits J Evol Biol 2001 14 892 903

[B19] Leinonen T O'Hara R Cano JM Merilä J Comparative studies of quantitative trait and neutral marker divergence: a meta-analysis J Evol Biol 2008 21 1 17 10.1111/j.1420-9101.2007.01445.x 18028355

[B20] Nottebohm F A brain for all seasons: cyclical anatomical changes in song control nuclei of the canary brain Science 1981 214 1368 1370 10.1126/science.7313697 7313697

[B21] Tramontin AD Brenowitz EA Seasonal plasticity in adult brain Trends Neurosci 2000 23 251 258 10.1016/s0166-2236(00)01558-7 10838594

[B22] Patel SN Clayton NS Krebs JR Spatial learning induces neurogenesis in the avian brain Behav Brain Res 1997 89 115 128 10.1016/s0166-4328(97)00051-x 9475620

[B23] Maguire EA Gadian DG Johnsrude IS Good CD Ashburner J Frackowiak RSJ Frith CD Navigation-related structural change in the hippocampi of taxi drivers Proc Nat Acad Sci 2000 97 4398 4403 10.1073/pnas.070039597 PMC18253 10716738

[B24] Marchettia MP Nevitt GA Effects of hatchery rearing on brain structures of rainbow trout, *Oncorhynchus mykiss* Env Biol Fish 2003 66 9 14

[B25] Kihslinger RL Nevitt GA Early rearing environment impacts cerebellar growth in juvenile salmon J Exp Biol 2006 209 504 509 10.1242/jeb.02019 16424100

[B26] Bănărescu PM Paepke HJ The Freshwater Fishes of Europe 2001 5/III AULA-Verlag, Wiebelsheim

[B27] Aiello LC Wheele P The expensive tissue hypothesis - The brain and digestive system in human and primate evolution Curr Anthrop 1995 36 199 221

[B28] Lande R Quantitative genetic analysis of multivariate evolution, applied to brain: body size allometry Evolution 1979 33 402 416 10.1111/j.1558-5646.1979.tb04694.x 28568194

[B29] Harvey PH Bennett PM Brain size, energetics, ecology, and life history patterns Nature 1983 306 314 315 10.1038/306314a0 6646214

[B30] Martin RD Harvey PH Jungers WL Brain size allometry: ontogeny and phylogeny Size and scaling in primate biology 1985 New York 147 173

[B31] Pagel MD Harvey PH The taxon-level problem in the evolution of mammalian brain size: facts and artifacts Am Nat 1988 132 344 359

[B32] Striedter GF Principles of Brain Evolution 2005 Sunderland: Sinauer Associates

[B33] Bauchot R Platel R Ridet JM Brain-body weight relationship in *Selachii* Copeia 1976 2 305 310

[B34] Herczeg G Gonda A Merilä J Evolution of gigantism in nine-spined sticklebacks Evolution 2009 63 3190 3200 10.1111/j.1558-5646.2009.00781.x 19624722

[B35] Herczeg G Gonda A Merilä J Rensch's rule inverted - female-driven gigantism in nine-spined stickleback *Pungitius pungitius* J Anim Ecol 2010 79 581 588 10.1111/j.1365-2656.2010.01665.x 20202005

[B36] Pollen AA Dobberfuhl AP Scace J Igulu MM Renn SCP Shumway CA Hofmann HA Environmental complexity and social organization sculpt the brain in Lake Tanganyikan cichlid fish Brain Behav Evol 2007 70 21 39 10.1159/000101067 17389793

[B37] Bauchot R Bauchot ML Platel RRidet JM Brains of Hawaiian tropical fish; brain size and evolution Copeia 1977 1/1977 42 46

[B38] Conover DO Schultz ET Phenotypic similarity and the evolutionary significance of countergradient variation Trends Ecol Evol 1995 10 248 252 10.1016/S0169-5347(00)89081-3 21237029

[B39] Alho JS Herczeg G Söderman F Laurila A Jönsson KI Merilä J Increasing melanism along a latitudinal gradient in a widespread amphibian: local adaptation, ontogenic or environmental plasticity? BMC Evol Biol 2010 10 317 10.1186/1471-2148-10-317 PMC2978224 20964816

[B40] Gienapp P Teplitsky C Alho JS Mills JA Merilä J Climate change and evolution: disentangling environmental and genetic responses Mol Ecol 2008 17 167 178 10.1111/j.1365-294X.2007.03413.x 18173499

[B41] Reader SM Laland KM Social intelligence, innovation, and enhanced brain size in primates Proc Natl Acad Sci USA 2002 99 4436 4441 10.1073/pnas.062041299 PMC123666 11891325

[B42] Gonzalez-Voyer A Winberg Kolm N Distinct evolutionary patterns of brain and body size during adaptive radiation Evolution 2009 63 2266 2274 10.1111/j.1558-5646.2009.00705.x 19473380

[B43] Pravosudov VV Kitaysky AS Omanska A The relationship between migratory behaviour, memory and the hippocampus: an intraspecific comparison Proc Roy Soc B 2006 273 2641 2649 10.1098/rspb.2006.3624 PMC1635458 17002950

[B44] Köhler M Moyà-Solà S Reduction of brain and sense organs in the fossil insular bovid *Myotragus* Brain Behav Evol 2004 63 125 140 10.1159/000076239 14726622

[B45] Jones KE MacLarnon AM Affording larger brains: Testing hypotheses of mammalian brain evolution on bats Am Nat 2004 164 E20 E31 10.1086/421334 15266377

[B46] Dunbar RIM Shultz S Evolution in the social brain Science 2007 317 1344 1347 10.1126/science.1145463 17823343

[B47] Herczeg G Gonda A Merilä J Predation mediated population divergence in complex behaviour of nine-spined stickleback (*Pungitius pungitius*) J Evol Biol 2009 22 544 552 10.1111/j.1420-9101.2008.01674.x 19210595

[B48] Herczeg G Gonda A Merilä J The social cost of shoaling covaries with predation risk in nine-spined stickleback (*Pungitius pungitius*) populations Animal Behav 2009 77 575 580

[B49] Birse SC Leonard RB Coggeshall RE Neuronal increase in various areas of the nervous system of the guppy, *Lebistes* J Comp Neurol 1980 194 291 301 10.1002/cne.901940202 7440802

[B50] Raymond PA Easter SS Jr Postembryonic growth of the optic tectum in goldfish. I. Location of germinal cells and numbers of neurons produced J Neurosci 1983 3 1077 1091 10.1523/JNEUROSCI.03-05-01077.1983 PMC6564515 6842282

[B51] Zupanc GKH Horschke I Proliferation zones in the brain of adult gymnotiform fish--a quantitative mapping study J Comp Neurol 1995 353 213 233 10.1002/cne.903530205 7745132

[B52] Zupanc GKH Adult neurogenesis and neuronal regeneration in the central nervous system of teleost fish Brain Behav Evol 2001 58 250 275 10.1159/000057569 11978945

[B53] Zupanc GKH Neurogenesis and neuronal regeneration in the adult fish brain J Comp Physiol A 2006 192 649 670 10.1007/s00359-006-0104-y 16463148

[B54] Kruska D Jerison HJ, Jerison I Mammalian domestication and its effect on brain structure and behavior Intelligence and Evolutionary Biology 1988 Springer-Verlag, Berlin 211 250

[B55] Kihslinger RL Lema SC Nevitt GA Environmental rearing conditions produce forebrain differences in wild Chinook salmon *Oncorhynchus tshawytscha* Comp Biochem Physiol A 2006 145 145 151 10.1016/j.cbpa.2006.06.041 16890467

[B56] Burns JG Saravanan A Rodd FH Rearing environment affects the brain size of guppies: lab-reared guppies have smaller brains than wild-caught guppes Ethology 2009 115 122 133

[B57] Eronen M Gluckert G Hatakka L van der Plassche O van der Plicht J Rantala P Rates of Holocene isostatic uplift and relative sea-level lowering of the Baltic in SW Finland based on studies of isolation contacts Boreas 2001 30 17 30

[B58] Herczeg G Turtiainen M Merilä J Morphological divergence of North-European nine-spined sticklebacks (*Pungitius pungitius*): signatures of parallel evolution Biol J Linn Soc 2010 101 413 416

[B59] Rohlf FJ 2002 tpsDig, digitize landmarks and outlines, version 1.37 Department of Ecology and Evolution, State University of New York at Stony Brook

[B60] Huber R van Staaden M Kaufman LS Liem KF Microhabitat use, trophic patterns and the evolution of brain structure in African cichlids Brain Behav Evol 1997 50 167 182 10.1159/000113330 9288416

