# Supplementary material for: Population variation in brain size of nine-spined sticklebacks (Pungitius pungitius) - local adaptation or environmentally induced variation?
Source: BMC Evol Biol. 2011 Mar 24;11:75. doi: 10.1186/1471-2148-11-75 (PMC3072340; doi:10.1186/1471-2148-11-75)
Supplement: Additional file 1 — Body size of the nine-spined sticklebacks (Pungitius pungitius) used in this study. Standard length and body weight of the nine-spined stickleback (Pungitius pungitius) individuals used in the present study in the different populations. Mean ± SD and the minimum - maximum range are presented. [file 1471-2148-11-75-S1.DOC]

**Additional file 1.** Body size of the nine-spined sticklebacks (*Pungitius pungitius*) used in this study. Standard length (measured from the tip of the nose to the end of the tailbase; SL) and fresh body weight (BW) are given. Mean ± Standard Deviation (SD) and the minimum – maximum range (in parentheses) are presented. N = 15 for every population. Underlined population code denotes common garden data. For the population abbreviations, see Fig. 1.

| Habitat | Population | SL (mm) | BW (g) |
| --- | --- | --- | --- |
| Marine | BÖL | 51.64 ± 2.51  (47.46 – 55.17) | 1.19 ± 0.22  (0.87 – 1.61) |
|  | HEL | 50.70±4.13  (41.63 – 57.96) | 1.11 ± 0.25  (0.71 – 1.63) |
|  | LEV | 50.93 ± 4.27  (44.23 – 57.61) | 1.16 ± 0.35  (0.71 – 1.93) |
|  | HEL | 44.89 ± 2.82  (40.56 – 49.2) | 0.9 ± 0.14  (0.61 – 1.09) |
|  | LEV | 54.67 ± 2.31  (50.22 – 58.34) | 1.41 ± 0.13  (1.18 – 1.67) |
| Lake | POR | 54.2 ± 1.41  (51.42 ­ 56.91) | 1.03 ± 0.09  (0.85 – 1.23) |
| Pond | BYN | 55.48 ± 2.99  (50.71 – 61.48) | 1.88 ± 0.31  (1.47 – 2.71) |
|  | PYÖ | 80.67 ± 5.54  (72.04 – 92.43) | 4.14 ± 0.9  (2.95 – 6.42) |
|  | RYT | 91.61 ± 2.55  (87.45 – 95.68) | 5.9 ± 0.51  (5.02 – 6.77) |
|  | MAS | 61.12 ± 2.99  (57.08 – 66.59) | 1.93 ± 0.21  (1.47 – 2.35) |
|  | BYN | 53.62 ± 3.45  (47.18 – 58.78) | 1.91 ± 0.38  (1.26 – 2.64) |
|  | PYÖ | 57.56 ± 5.08  (51.12 – 69.96) | 2.04 ± 0.57  (1.16 – 3.35) |
